# cGMP production of patient-specific iPSCs and photoreceptor precursor cells to treat retinal degenerative blindness

**DOI:** 10.1038/srep30742

**Published:** 2016-07-29

**Authors:** Luke A. Wiley, Erin R. Burnight, Adam P. DeLuca, Kristin R. Anfinson, Cathryn M. Cranston, Emily E. Kaalberg, Jessica A. Penticoff, Louisa M. Affatigato, Robert F. Mullins, Edwin M. Stone, Budd A. Tucker

**Affiliations:** 1 Stephen A. Wynn Institute for Vision Research, Department of Ophthalmology and Visual Sciences, Carver College of Medicine, University of Iowa, Iowa City, IA, USA

## Abstract

Immunologically-matched, induced pluripotent stem cell (iPSC)-derived photoreceptor precursor cells have the potential to restore vision to patients with retinal degenerative diseases like retinitis pigmentosa. The purpose of this study was to develop clinically-compatible methods for manufacturing photoreceptor precursor cells from adult skin in a non-profit cGMP environment. Biopsies were obtained from 35 adult patients with inherited retinal degeneration and fibroblast lines were established under ISO class 5 cGMP conditions. Patient-specific iPSCs were then generated, clonally expanded and validated. Post-mitotic photoreceptor precursor cells were generated using a stepwise cGMP-compliant 3D differentiation protocol. The recapitulation of the enhanced S-cone phenotype in retinal organoids generated from a patient with *NR2E3* mutations demonstrated the fidelity of these protocols. Transplantation into immune compromised animals revealed no evidence of abnormal proliferation or tumor formation. These studies will enable clinical trials to test the safety and efficiency of patient-specific photoreceptor cell replacement in humans.

Heritable retinal degenerative disorders, such as retinitis pigmentosa (RP), Stargardt disease, and Leber congenital amaurosis, are a major cause of incurable blindness worldwide. Vision loss associated with these diseases results from death of the light sensing photoreceptor cells of the outer neural retina. Fortunately, in the majority of retinal degenerative patients, the inner layers of the neural retina that functionally connect the photoreceptors to the brain remain relatively intact^1^,^2^. This, coupled with the fact that the retina does not contain inhibitory myelin-associated proteins found in other CNS compartments, makes transplantation-based photoreceptor cell replacement an attractive treatment strategy for the restoration of visual function.

A variety of different cell types, ranging from retinal progenitor cells^3^,^4^,^5^ isolated from developing fetuses to mature photoreceptor cells isolated from post-mortem donor eyes^6^, have been tested in retinal degenerative models for the ability to restore retinal function. Collectively, these experiments revealed the post-mitotic photoreceptor precursor cell to have the greatest capacity to survive, integrate with the remaining host retina and develop into mature functional photoreceptor cells following transplantation^4^,^7^,^8^,^9^,^10^,^11^. Although it is not feasible to obtain photoreceptor precursor cells from human donor tissue for clinical applications due to the differentiation state of these cells and post-mortem degradation, recent advancements in pluripotent stem cell technology have made it possible for scientists to generate these cells under controlled conditions as needed. For instance, many groups, including our own, have demonstrated the ability to use pluripotent stem cells to derive functional photoreceptor precursors that have the ability to restore retinal structure and function in animals following transplantation into retinal degenerative hosts^7^,^9^,^10^,^12^,^13^,^14^,^15^,^16^,^17^,^18^,^19^,^20^,^21^.

To date, most pluripotent stem cell-derived photoreceptor precursor cells have been generated using either: (1) an adherent 2D culture system in which exogenous factors known to drive forebrain and eye field development are administered^3^,^4^,^7^,^16^,^22^,^23^,^24^, or (2) a floating 3D culture system that couples the cells’ intrinsic ability to spontaneously differentiate and self-organize with the experimenters’ ability to positively identify and enrich for the desired tissue types^21^,^25^,^26^,^27^,^28^. There are inherent advantages and disadvantages of each of these different approaches. For instance, the 2D system is well suited for testing drug and gene augmentation therapies in which widespread cellular targeting is required. It is much more difficult to transduce a significant number of cells within floating 3D organoids^29^. The 3D culture system on the other hand is more amenable to cellular enrichment and as such is the ideal system for development of a tissue-specific transplantation strategy. With the 2D system, it is difficult to obtain a sufficient population of cells for transplantation without targeted cell sorting (e.g., FACS) or magnetic bead panning which are often both harsh and inefficient^7^,^30^.

Despite the experimental utility of embryonic stem cell (ESC)- and induced pluripotent stem cell (iPSC)-derived retinal cells, there is some debate about which stem cell type is most clinically relevant. There are ethical concerns associated with the harvesting of embryonic tissues needed for generation of ESCs as well as immunological challenges associated with transplanting cells into unmatched recipients. It is likely that retinal degeneration patients treated with photoreceptor precursor cells derived from ESCs will require prolonged immunosuppressive therapy. Both of these issues can be overcome with patient-specific, autologous iPSCs. Specifically, when paired with genome editing, iPSC methods allow one to obtain genetically normal, immunologically-matched cells for retinal transplantation without using any embryonic or fetal tissue. Although an iPSC strategy would likely obviate the need for life long immunosuppression, the patient-specific approach is not without its drawbacks. Specifically, ESCs, can be validated, grown and differentiated in sufficient quantities to treat a large number of patients, while autologous iPSCs must be generated and validated on a per patient basis. The latter approach is therefore costlier and requires a larger number of dedicated highly skilled personnel per treated patient and thus may not be commercially viable in the foreseeable future. However, if xeno-free, current Good Manufacturing Practice (cGMP)-compatible iPSC protocols could be developed and shared freely, the approach could be realistically employed in non-profit academic centers that serve regional patient populations.

In this study we report the successful development of xeno-free, cGMP-compliant protocols for the production of clinical-grade iPSCs and photoreceptor precursor cells regardless of patient age, sex, or genotype. Specifically, we have successfully isolated and expanded patient-specific dermal fibroblasts, generated iPSCs and used them to derive recoverin-, NRL-, CRX-, and NR2E3-positive, photoreceptor precursor cells in an ISO class 5 environment.

## Materials and Methods

### Patients

All patients provided written, informed consent for this study, which was approved by the Institutional Review Board of the University of Iowa (project approval #199904167) and adhered to the tenets set forth in the Declaration of Helsinki.

### Quality control testing pre-cGMP release

Any reagents determined to have insufficient certificate of analysis information with respect to human pathogens (i.e., such as CytoTune-iPS 2.0 which is sterile and xeno-free but is provided as non-commercial research grade only) are lot tested as outlined in Table 1. Data is subsequently reviewed by the quality assurance director and the product is released to the cGMP facility.

### Isolation of Fibroblasts from Patient Dermal Biopsies

3 mm skin biopsies were obtained from either the non-sun-exposed upper arm or lower abdomen and minced in Iowa xeno-free biopsy media, IxMedia. IxMedia consists of 395 mL MEM-alpha (Gibco/Thermo Fisher Scientific, Grand Island, NY; Cat#: 12571-063;), 50 mL CTS KnockOut Serum Replacement XenoFree Medium (Gibco/Thermo Fisher Scientific; Cat#: 12618013), 50 mL Heat-Inactivated Human Serum (Innovative Research, Novi, MI; Cat#: IPLA-SERAB-HI, lot tested for potency and analyzed as per Table 1 prior to release for cGMP use), 5 mL GlutaMAX Supplement (Gibco/Thermo Fisher Scientific; Cat#: 35050-061), 1 mL Primocin (InvivoGen, San Diego, CA; Cat#: ant-pm-2) and 10 ng/mL rhFGF2 cGMP Grade (Waisman Biomanufacturing, Madison, WI; Cat#: rhFGF). After mincing, tissue fragments were allowed to adhere to 6-well tissue culture-treated plates via air drying, and then cultured in IxMedia at 5% CO_2_, 20% O_2_, 37 **°**C. These cultures were fed every other day with 2 mL of fresh IxMedia per well. For the first 2 feedings, 1% ECM mixture ((human type 1 and type 3 collagen/vitronectin/fibronectin, Advanced BioMatrix, Carlsbad, CA; collagen type 1 (Cat#: 5007-20 ML), collagen type 3 (Cat#: 5021-MG), vitronectin (Cat#: 5051-0.1MG) and fibronectin (Cat#: 5050-1MG), mixture tested as per Table 1 prior to release and cGMP use) was added to the media. Once cells reached confluence, they were passaged using xeno-free TrypLE Express (Life Technologies/Thermo Fisher Scientific, Grand Island, NY; Cat#: 12604-013) and used for generation of patient-specific induced pluripotent stem cells (iPSCs).

### Patient-specific iPSC generation

**Figure 1:**
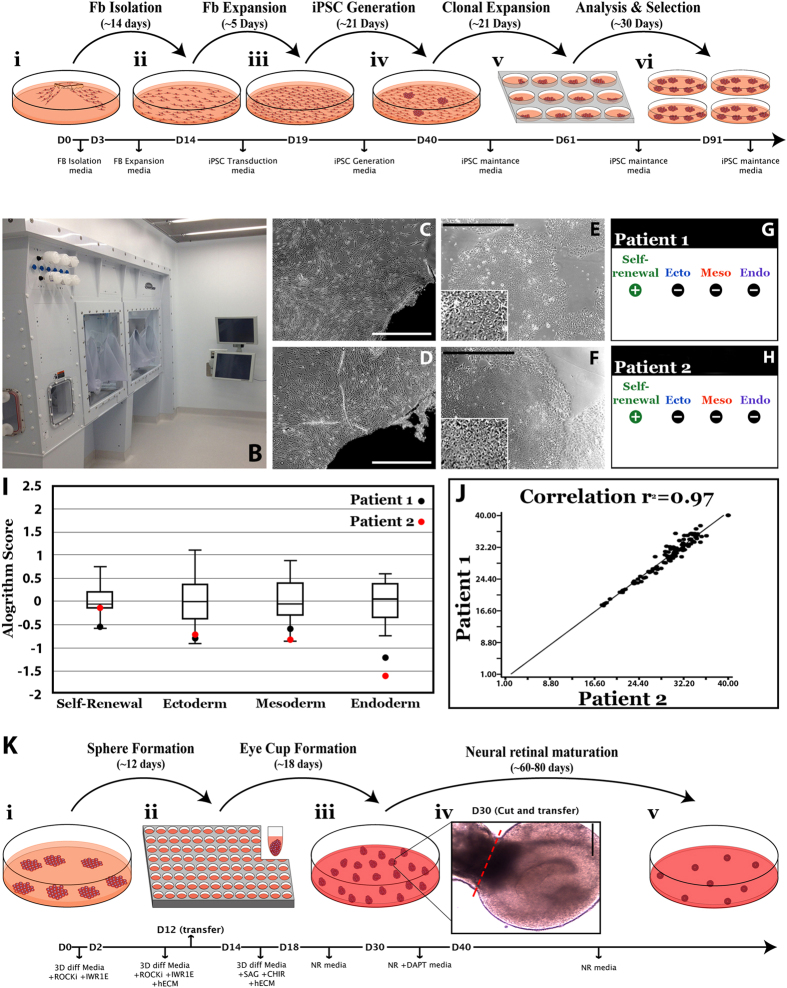
Generation of cGMP, clinical-grade patient-specific iPSCs. (**A**) Schematic depicting the timeline and stepwise procedure for cGMP-compliant fibroblast isolation from patient skin biopsies and subsequent generation, clonal expansion and analysis of patient-derived iPSCs. (**B**) Photograph of one of two cGMP processing suites within the Steven W. Dezii Translational Vision Research Facility equipped with an ISO class 5 BioSpherix Xvivo Closed Incubation System. (**C–F**) Light micrographs of two independent patient-derived cell lines (DB-005 (**C**,**E**) and DB-006 (**D**,**F**)). Fibroblasts can be seen migrating from and growing around a fragment of patient skin (**C**,**D**). Typical iPSC colonies with large nuclear to cytoplasm ratio generated from each fibroblast line are also shown (**E,F** and insets in each). (**G,H**) Data generated by hPSC Scorecard Analysis Software demonstrating each independent patient line primarily expresses genes that participate only in self-renewal, not in formation of endoderm, mesoderm or endoderm. (**I)** Graph comparing the algorithm scores for expression of genes involved in self-renewal. Compared to the internal reference data set provided by the software, each patient line falls within the average. (**J)** Graph showing the correlation coefficient comparing all gene expression data for each patient line. The two lines are highly correlated to one another (r^2^ = 0.97). These data demonstrate how similar each line is to one another, speaking to the consistency of our cGMP protocol for the generation of patient-specific iPSC lines. (**K)** Schematic showing the timeline and stepwise procedure for cGMP-compliant three-dimensional differentiation of patient iPSCs and the production of iPSC-derived retinal organoids.

250,000 patient-specific dermal fibroblasts were plated in 1 well of a 6-well culture dish with IxMedia. The following day (24 hours prior to transduction) media was switched to xeno-free, serum-free, IxMedia. The following day, cells were transduced with non-integrating Sendai viral vectors^10^,^22^,^31^,^32^ driving expression of *OCT4*, *SOX2*, *KLF4* and *c-MYC* at an MOI of 3 (Invitrogen/Thermo Fisher Scientific, Waltham, MA; CytoTune-iPS 2.0 Sendai Reprogramming Kit; Cat#: A16517, as indicated above and previously by Schlaeger *et. al.*, CytoTune-iPS 2.0 is licensed for research purposes only, as such for commercial/clinical use further licensing maybe required^33^. As indicated above, prior to release for cGMP use, an aliquot of the CytoTune-iPS 2.0 Sendai Reprogramming Kit, which was isolated from a master lot demonstrated to be effective for reprogramming, was scrutinized as per Table 1 and determined to be free from tested contaminants.) in Viral Transduction Media (serum free IxMedia plus 10 μM Y-27632 ROCK Inhibitor (EMD Milipore, Billerica, MA; Cat#: 688000)). Transduction media was removed 18 hours later and replaced daily with serum free IxMedia. Five days post-transduction, fibroblasts were passaged onto fresh xeno-free rhLaminin 521 (LN521)-coated 10 cm culture dishes (Corning Life Science, Tewksbury, MA; Cat#: 354222) and fed with fresh serum free IxMedia plus Revita Cell (Life Technologies/Thermo Fisher Scientific; Cat# A26445-01). On day 6, the media was transitioned to xeno-free Human Essential 6 (Life Technologies/Thermo Fisher Scientific; Cat#: A1516401) using equal parts Essential 6 and serum free IxMedia. From day 7 to day 21 cultures were fed daily with fresh Essential 6. On day 21, cultures were transitioned to Human Essential 8 iPSC maintenance media (Life Technologies/Thermo Fisher Scientific; Cat#: A1517001) and fed daily. When iPSC colonies reached 1–2 mm in diameter they were manually isolated and passaged on fresh 12-well rhLaminin521-coated culture plates and clonally expanded in Human Essential 8 media. Following expansion, cells were analyzed for pluripotency (i.e., expression of endogenous pluripotency factors) via rt-PCR and loss of transgene expression (i.e. lack of detectable expression of *OCT4*, *SOX2*, *KLF4* or *c*-MYC) using a qPCR-based scorecard assay^34^,^35^. A summary of this protocol is depicted in Fig. 1A.

### TaqMan Human Pluripotent Stem Cell Scorecard Panel

Pluripotency of patient-derived iPSCs was assessed using the TaqMan Human Pluripotent Stem Cell Scorecard Panel (Life Technologies/Thermo Fisher Scientific; Cat #: A15870 and Cat # A15872)^34^,^35^. Total RNA was isolated using the RNeasy Mini kit (Qiagen) according to manufacturer’s instructions. One microgram of RNA was reverse transcribed using the Superscript VILO cDNA Synthesis Kit (Life Technologies/Thermo Fisher Scientific; Cat #: 11754050). cDNA was added to an hPSC Scorecard Plate (Life Technologies/Thermo Fisher Scientific; Cat #: A15870) and amplified using a QuantStudio 6 Flex Real-time PCR System with 384-well capability (Life Technologies/Thermo Fisher Scientific). Gene expression data were uploaded to the web-based hPSC Scorecard Analysis Software (Life Technologies/Thermo Fisher Scientific) for interpretation.

### Karyotyping of iPSC Lines

iPSC lines were karyotyped using a standard G-banding protocol. Briefly, cells were arrested at metaphase with 0.02 mg/mL colcemid (Sigma-Aldrich, St. Louis, MO; Cat#: 7385) and the chromosomes stained using Giemsa (Sigma-Aldrich, St. Louis, MO; Cat# GS500). The chromosomal number of each line was determined by microscopic analysis and the cells were examined for the presence or absence of detectable structural rearrangements. At least 5 cells from each line were analyzed and 3 cells from each line karyotyped.

### Whole genome sequencing of patient-derived cells

A common concern associated with cellular reprograming and *in vitro* expansion is introduction of harmful mutations. To measure this, we conducted whole genome sequencing on two individuals using DNA extracted from peripheral blood, fibroblasts and 3 independent clones of iPSCs. Sequencing was conducted on an Illumina HiSeqX to an average depth exceeding 30× for each of the ten samples. Sequence was aligned to the genome using BWA and variants were called simultaneously using the GATK HaplotypeCaller. Variants were filtered to remove those with a QD < 5, those > 0.1% in control populations, (ExAC, EVS and 1000 genomes) and to remove those variants found in a database of internal control genomes processed using the same methods (n = 36).

### Patient-specific retinal cell generation

To generate retinal cells from patient-specific iPSCs, cells were differentiated using clinical-grade, xeno-free, cGMP-validated reagents using a 3-dimensional (3D) suspension culture protocol. Specifically, cell culture media was aspirated from iPSCs, and the cells were incubated in 1 mL of xeno-free TrypLE Express (Life Technologies/Thermo Fisher Scientific) for 3 minutes at 37 °C. Following incubation, the side of the plate was vigorously tapped to release the cells and a 1 mL pipette was used to gently flush the cells from the plate. The cells were transferred into a 50 mL centrifuge tube. The wells were rinsed with 2 mL of 3D differentiation media [DMEM (Gibco/Thermo Fisher Scientific; Cat#: 11965-118), 10% heat-inactivated human serum (Innovative Research), 20% CTS KnockOut Serum Replacement XenoFree medium (Gibco/Thermo Fisher Scientific; Cat#: 12618013), 0.1 mM MEM non-essential amino acids (Gibco/Thermo Fisher Scientific; Cat#: 11140-050), 1 mM Sodium Pyruvate (Gibco/Thermo Fisher Scientific; Cat#: 11360-070), 0.1 mM 2-Mercaptoethanol (Gibco/Thermo Fisher Scientific; Cat#: 21985-023), 1% ECM (human type 1 and type 3 collagen/vitronectin/fibronectin, Advanced BioMatrix), 20 mM Y-27632 ROCK Inhibitor (EMD Milipore), 3 nM IWR1e (Cayman Chemical, Ann Arbor, MI; Cat#: 13659), 3 M StemMACS CHIR (Miltenyi Biotec Inc., San Diego, CA; Cat#: CHIR99021), 100 nM SAG (Enzo Life Sciences, Farmingdale, NY, Cat#: ALX-270-426)] per well and this was added to the same 50 mL tube. After centrifuging this tube at 200× g for 3 minutes, the supernatant was aspirated and discarded and the cells were resuspended in fresh 3D differentiation media. The cells were then counted using a Scepter 2.0 cell counter (EMD Millipore) and plated on 96-well ultra low adhesion sphere forming plates (Corning Life Sciences; Cat#: 7007). For each 96-well plate, 1 × 10^6^ cells were added to 10 mL of 3D differentiation media with ROCKi and IWR1e. Using a multichannel pipettor, 1 × 10^4^ cells (100 μL of the suspension) were added to each well. The cells were then incubated at 37 **°**C with 5% CO_2_ for two days. At this point, 100 μL of 3D differentiation media supplemented with ROCKi, IWR1e and 1% ECM was added to each well. Six and 10 days after plating, the multichannel pipettor was used to remove 100 μL of media from each well and replace it with 100 μL of fresh media (using the same formula as day 2). On day 12, the spheres were transferred to 100 mm ultra-low attachment cell culture dishes (Corning Life Sciences; Cat#: 3262) at a density of one 96-well plate per 100 mm dish. The media was removed and replaced with fresh 3D differentiation media supplemented with 1% ECM. The cultures were fed every other day from this point forward. On days 14–17, the spheres were fed with 3D differentiation media supplemented with 1% ECM, 3 mM CHIR and 100 nM SAG. On day 18, the 3D differentiation media was removed and switched to neural retina culture medium [CTS KnockOut DMEM/F12 (Gibco/Thermo Fisher Scientific; Cat#: A1370801), 2 mM GlutaMAX supplement (Gibco/Thermo Fisher Scientific; Cat#: 35050061), and CTS N-2 supplement (Cell Therapy Systems, Thermo Fisher Scientific, Grand Island, NY; Cat#: A1370701)]. On days 30–40, 10 mM DAPT (EMD Millipore; Cat#: 565770) was added to enhance photoreceptor cell production. The cells were then fed three times per week with neural retina media until harvest. A summary of this protocol is depicted in Fig. 1K.

### Immunocytochemistry of iPSC-derived organoids

3D iPSC-derived organoids were embedded in 4% agarose and sectioned at a thickness of 100 μm using a Leica VT1000 S vibratome (Leica Microsystems, Wetzlar, Germany) and stained with the following antibodies: mouse anti-SOX2 (R&D Systems, Minneapolis, MN; 1:1000; Cat#: MAB2018), rabbit anti-PAX6 (BioLegend, San Diego, CA; 1:1000; Cat#: PRB-278 P), goat biotinylated-anti-OTX2 (R&D Systems; 1:1000; Cat#: BAF1979), rabbit anti-Ki67 (Abcam, Cambridge, MA; 1:500; Cat#: ab15580), sheep anti-VSX2/CHX10 (Exalpha Biologicals, Shirley, MA; 1:100; Cat#: X1180P), mouse anti-MITF (Exalpha Biologicals; 1:500; Cat#: X1405M), mouse anti-HuC/D (Life Technologies/Thermo Fisher Scientific; 1:500; Cat#: A-21272), rabbit anti-TUJ1 (neuron-specific class III beta-tubulin; Sigma-Aldrich, St. Louis, MO; 1:1000; Cat#: T2200), goat biotinylated-anti-NRL (R&D Systems; 1:250; Cat#: BAF2945), mouse anti-LAMP-1 (Abcam; 1:250; Cat#: ab25630), mouse anti-Rhodopsin (EMD Millipore; 1:1000; Cat#: MAB5356), rabbit anti-SW Opsin (EMD Millipore; 1:1000; Cat#: AB5407), rabbit anti-red/green(L/M)-Opsin (EMD Millipore; 1:1000; Cat#: AB5405), sheep anti-CRX (R&D Systems; 1:100; Cat#: AF7085), rabbit anti-Recoverin (EMD Millipore; 1:2000; Cat#: AB5585), mouse anti-TRA-1-81 (EMD Millipore; 1:1000; Cat#: MAB4381), mouse anti-TRA-1-60 (EMD Millipore; 1:1000; Cat#: MAB4360), rabbit anti-SSEA-3 (EMD Millipore; 1:1000; Cat#: MAB4303), and mouse anti-SSEA-4 (EMD Millipore; 1:1000; Cat#: MAB4304). To label filamentous actin (F-actin), Alexa Fluor 488 Phalloidin (Life Technologies/Thermo Fisher Scientific; 1:2000; Cat#: A12379) was used. Primary antibodies were detected using the species appropriate, fluorescently conjugated Alexa Fluor secondary antibodies [Life Technologies/Thermo Fisher Scientific; 1:1000; goat anti-mouse 488 (Cat#: A-11001), goat anti-rabbit 568 (Cat#: A-11011), Streptavidin 647 (Cat#: S-21374) and donkey anti-sheep 647 (Cat#: A-21448)]. Cell nuclei were counterstained using DAPI. Organoid thick sections were imaged using a Leica DM 2500 SPE confocal microscope (Leica Microsystems).

### Quantification of retinal organoid production efficiency

To determine the efficiency of retinal organoid formation, the number of spheres in each 96-well sphere forming plate that developed retinal epithelial protrusions suitable for isolation and expansion was counted. In this experiment 6 independent patient-specific cell lines were used and the differentiation experiment was repeated 2 independent times.

### Quantification of SW Opsin-positive cells in retinal organoids

Organoids derived from two patients with *NR2E3*-associated enhanced S-cone syndrome (NR2E3-1 and NR2E3-2) and from an unaffected control patient with normal ocular history were assessed for positive immunolabeling for SW Opsin (EMD Millipore; 1:1000; Cat#: AB5407) at 15–16 weeks post-differentiation. At least 250 cells were assessed per group and the percentage of SW Opsin-positive cells observed for each was calculated. Cell nuclei were counterstained using DAPI.

### Final product release criteria

Prior to being released from the cGMP facility for downstream applications, patient-specific cells must meet the release criteria described in Table 2. These criteria are developed to demonstrate both cellular identity and sterility. If it is determined that a single cell type is most efficient for retinal transplantation, cellular purification can be performed via FACS or magnetic bead-based cell sorting, both of which have been shown to be efficient for downstream applications^36^,^37^.

### Assessment of tumor potential of iPSC-derived 3D organoids

iPSC-derived 3D organoids, 13–16 weeks post-differentiation were dissociated, pooled and 1–5 × 10^6^ cells per 100 μL of sterile PBS were injected intramuscularly into the hind limbs of immunocompromised SCID mice (Jackson Laboratories, Bar Harbor, ME; Stock No. 001303). Mice were evaluated for tumor formation at 3 months post-injection. To confirm lack of tumor formation, two independent long-term injection studies were carried out for over 10 months in two cohorts of 4 mice each. Additionally, ten SCID mice received a single subretinal injection of 1 × 10^6^ cells in sterile PBS. Mice were evaluated funduscopically for intra-ocular tumors at 3-months post-injection.

### Statistical Analysis

One-way ANOVA with a Tukey’s posthoc test for multiple comparisons was used to determine significance between *NR2E3* and control patients using Prism 6 (GraphPad Software, Inc., La Jolla, CA). P < 0.05 was considered statistically significant.

## Results

To develop a reusable autologous iPSC-based cell replacement approach for the treatment of inherited retinal degenerative blindness, two major steps were required: (1) the design and construction of a dedicated non-profit cGMP facility that could be readily replicated by other academic institutions, and (2) the development of cGMP-compliant processes for manufacture of photoreceptor precursor cells. To address the first of these two points, using philanthropic support, we recently designed, built and certified a small non-profit cGMP facility that is dedicated to the production of gene- and cell-based therapeutics for the treatment of inherited retinal degenerative blindness (Steven W. Dezii Translational Vision Research Facility). This facility contains an independent HEPA-filtered equipment room (Supplemental Fig. 1A), an ISO Class 7 (Class 10,000) material storage and handling room (Supplemental Fig. 1B), 2 ISO Class 7 (Class 10,000) gowning areas (Supplemental Fig. 1C), 2 ISO Class 6 (Class 1000) processing rooms (Fig. 1B, Supplemental Fig. 1D) and an unclassified service chase with dedicated access for equipment and facility maintenance (Supplemental Fig. 1F). Each of the two processing rooms are equipped with custom BioSpherix Xvivo Closed Incubation Systems, which are designed to exceed ISO Class 5 (Class 100) cleanliness requirements to ensure minimal biological contamination throughout standard operating procedures, while providing absolute optimal conditions for the long-term culturing of cells under defined atmospheric conditions (Fig. 1B, Supplemental Fig. 1E).

### cGMP isolation and expansion of aged, patient-specific dermal fibroblasts

To ensure the safety of participants in clinical trials and to meet the requirements of federal regulatory agencies, human cell-based therapeutics must be generated in compliance with cGMP guidelines for the manufacture of Human Cells, Tissues, and Cellular and Tissue-Based Products (HCT/P’s; www.fda.gov). It has been shown that cells exposed to animal products have the potential to incorporate immunogenic antigens^38^,^39^,^40^,^41^. Unlike viral vectors, which can be physically purified from all production components, animal-derived products must be completely excluded from the manufacture of autologous iPSC-derived therapeutics to maintain a perfect immunological match.

Although reprogramming protocols designed to generate patient-specific iPSCs using dermal fibroblasts have largely used cells grown under non-xeno-free conditions (i.e. fibroblast cell culture media containing fetal bovine serum (FBS)), fully validated, xeno-free fibroblast cell culture media are commercially available, including FibroGRO (Millipore), FGM-CD (Lonza), and MesenCult (Stemcell technology). It is likely that the initial phase 1 safety trials of photoreceptor precursor transplantation will occur in patients with very advanced disease who have light perception or poorer visual acuity. The majority of retinal degeneration patients with this degree of vision loss are older than 55 years, and we therefore thought it important to explore the effect of patient age on the success of our cGMP protocols.

As the majority of patients that would be eligible for a phase 1 safety study, i.e. have advanced disease and visual acuity of light perception or poorer, are elderly we decided to test the commercially available reagents in our cGMP facility using biopsies isolated from one of our eldest patients (81 years-of-age). We began by using FibroGRO™, a reagent that was very recently reported by Wang and colleagues to be useful for the isolation and expansion of foreskin fibroblasts suitable for iPSC generation^42^, to isolate and expand a biopsy from an 81-year-old completely blind individual with RP. Unfortunately, using the standard biopsy isolation and fibroblast culture protocol described in the methods section above, we were unable to obtain fibroblast cells from this patient. As it was possible that the lack of proliferation of an aged patient’s cells in the cGMP protocol was a patient-specific event, we repeated this experiment using skin biopsies obtained from 7 individuals ranging from 29–81 years of age (Table 3). Although FibroGRO was useful for the isolation and expansion of cells from the youngest patient in the cohort (i.e., we were able to obtain a sufficient number of cells for iPSC generation from the 29-year-old donor (Table 3)), the number of fibroblasts obtained decreased with patient age and we could not obtain any fibroblasts under these conditions from individuals beyond 47 years of age (Table 3). To further explore this age effect, FGM-CD and MesenCult were evaluated for their ability to expand fibroblasts that were previously isolated using traditional media containing 10% FBS. For these experiments, two separate patient cohorts, one in the 7^th^ decade of life and a second in the 8^th^ decade of life, were used. In addition to the commercially available media, an in-house-generated xeno-free medium (IxMedia) was used. This medium (completely specified in Methods above) was designed by replacing FBS, the main component in non-cGMP media, with human-qualified cGMP grade heat inactivated human serum and knockout serum replacement. In addition, cGMP grade bFGF was added to promote cellular proliferation and a 1% ECM mixture was added for the first 48 hours post-plating to promote cellular adhesion.

Although cultures fed with FGM-CD or MesenCult were sparse, some dermal fibroblasts with typical morphology could be identified 10 days after plating (Supplemental Fig. 2A–D). Interestingly, even though an equal number of cells were plated in each condition, there were more healthy cells evenly distributed across the culture surface 1 day after plating in IxMedia than there were 10 days after plating in either FGM-CD or MesenCult (Supplemental Fig. 2E,F). Ten days after plating, cultures fed with IxMedia were confluent and required passaging (Supplemental Fig. 2G,H). To further assess the utility of IxMedia for cGMP fibroblast isolation and expansion, an additional biopsy was obtained from the same 81-year-old patient used to test FibroGRO above and was cultured in IxMedia. Extensive outgrowth was detected seven days after plating (Supplemental Fig. 2I) and by 14 days, cultures were confluent and ready to be passaged (Supplemental Fig. 2J). To further evaluate the robustness of IxMedia for fibroblast isolation and expansion, biopsies were obtained from an additional 34 individuals, ranging from 17–86 years of age (Table 4). Each of these patients had been diagnosed with an inherited retinal degenerative disease and 30 of them had received molecular confirmation of their disease-causing mutations (Table 4). Of these, 28 patients had vision of light perception or worse in their poorer eye and were therefore suitable for inclusion in a phase 1 cell replacement trial (Table 4).

### cGMP production of patient-specific iPSCs

Following fibroblast isolation and expansion, we sought to develop a cGMP-compatible method for iPSC generation. To do so, we adopted aspects of our previously published feeder-free/xeno-free iPSC generation protocol^22^, which uses non-integrating footprint-free Sendai virus to drive expression of the transcription factors *OCT4*, *SOX2*, *KLF4* and *c-MYC* (Fig. 1A). For this series of experiments, we used two independent patient-specific fibroblast lines, DB-005 (Fig. 1C) and DB-006 (Fig. 1D), obtained from donors 47 and 32 years of age, respectively. Twenty-one days after transduction, 12 iPSC clones were selected from each line and expanded on LN521-coated 12-well cell culture plates in E8 media as described in the methods section above (Fig. 1A). The top 4 clones from each line, as determined by normal karyotype, cellular morphology and ability to be maintained in a pluripotent state (Fig. 1E,F). To look for deleterious variants that may be caused by Sendai virus-mediated reprogramming or cell line expansion, we conducted whole genome sequencing from peripheral blood, fibroblasts, and iPSCs from DB-005 (Fig. 1C) and DB-006 (Fig. 1D). Unique variant counts are presented in Supplemental Fig. 3. On average, each of the six iPSC clones assessed harbored 579 unique high-quality SNPs and 161 unique high-quality InDels. Of these there were an average of 8.2 non-silent exonic SNPs and 0.7 exonic InDels. None of these exonic variants fell in known cancer-specific genes annotated by the Cosmic gene census. These results are similar to those previously described for a Sendai virus-based reprogramming protocol^43^.

Following 10 passages, total RNA was isolated and pluripotency was assessed using the TaqMan Human Pluripotent Stem Cell Scorecard Panel^34^,^35^. This assay compares transcript expression levels in lines of interest to an undifferentiated iPSC reference gene set^34^,^35^. Real-time PCR analysis of genes required for self-renewal, ectoderm, mesoderm or endoderm formation was assessed on the Scorecard Panel. As expected, both patient-specific iPSC lines expressed genes that are solely involved in self-renewal (Fig. 1G,H). Further, Sendai viruses were not detected in any patient-specific iPSC line. A box plot comparing algorithm scores for genes involved in self-renewal for each line are shown in Fig. 1I. The algorithm score of each independently generated line falls within the mean algorithm score of the reference gene set and the expression of genes involved in self-renewal are similar between the lines (Fig. 1I). Furthermore, a scatter plot of pair-wise comparisons of all ΔCt values for genes tested showed that the two lines are transcriptionally very similar to one another (Fig. 1J; Patient 1 (DB-005) vs Patient 2 (DB-006) r^2^ = 0.97). Taken together, these data demonstrate that we have successfully isolated and expanded patient-specific fibroblasts and generated clinical-grade patient-specific iPSCs in a cGMP-compliant manner regardless of patient age.

### cGMP 3D derivation of photoreceptor precursor cells

As indicated above, the 3D differentiation system is well suited for the production of purified neural retinal cells. For example, using a protocol adapted from that of Nakano *et al*.^17^ we recently demonstrated how this system could be used to recapitulate retinal development and give rise to a large number of photoreceptor precursor cells suitable for the investigation of disease pathophysiology^32^. However, this protocol also required modification to be cGMP-compliant. Two of the major components of previously published 3D differentiation protocols that required replacement were the animal-derived products Matrigel (an extracellular matrix mixture extracted from Engelbreth-Holm-Swarm mouse sarcoma cells) and FBS, which are known to enhance neural epithelia formation and retinal genesis, respectively^17^,^44^. These reagents were replaced with cGMP grade human serum and a mixture of human extracellular matrix proteins, all of which were sourced from FDA-certified facilities. As with previously published 3D differentiation protocols, this method is based on the self-organization and physical selection of retinal organoids, which spontaneously develop in suspension^17^,^21^,^28^,^32^. As described in detail in the methods section, patient-specific iPSCs are passaged as a single cell suspension and cultured in 96-well ultra-low-adhesion sphere forming plates in 3D differentiation media containing the ROCK inhibitor Y-27632, the WNT inhibitor IWR1e and a 1% human ECM mixture (Fig. 1K-ii). Y-27632 was added to promote cell survival following single cell passage^17^,^45^. The ECM mixture was included to enhance neural epithelium formation^17^,^44^. IWR1e was added to combat the caudalization effects of KSR, which was required for sphere formation^17^,^46^. Twelve days after plating, spheres were transferred to ultra-low-adhesion 100 mm dishes (Fig. 1K-iii). At day 14, 3D differentiation media was supplemented with a 1% human ECM mixture, the Smoothened agonist SAG, which has been shown to enhance neural retinal formation^17^,^47^, and the GSK3β inhibitor CHIR, which when delivered transiently has been shown to promote retinal-pigmented epithelium (RPE) formation)^17^. On day 18, spheres were transferred to neural retina media and fed every other day until day 30. Cultures were subsequently fed every other day with neural retina media supplemented with the Notch inhibitor DAPT, which has been shown to promote photoreceptor cell genesis and suppress glial cell genesis^10^,^17^,^21^,^22^,^28^. Between days 30 and 40, spheres with clearly identifiable retinal cups and pigmented RPE were isolated and the organoids were dissected free from the remaining spheres (Fig. 1K-iv). On Day 40, DAPT was removed from the media and organoids were subsequently fed every other day with neural retina media until time of harvest (Fig. 1K).

**Figure 2:**
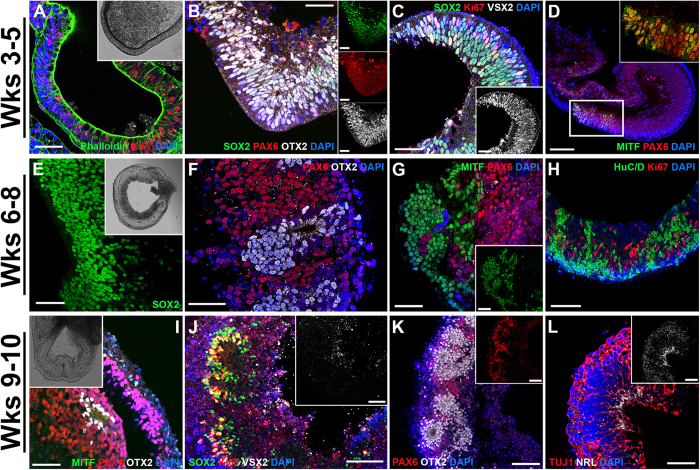
Early 3D organoid development in patient-specific iPSCs. Immunocytochemical analysis of 3D organoids after 3–5, 6–8 and 9–10 weeks of differentiation. (**A–D)** After 3–5 weeks of differentiation, 3D spheres have evaginated loops comprised of polarized F-actin-positive structures (**A** and inset; Phalloidin; green) and proliferating, Ki67-positive neural epithelia (**A**; red). At this stage, spheres express SOX2 (**B**; green), PAX6 (**B**; red) and OTX2 (**B**; gray) and have the appearance of developing organoids. Many cells positive for SOX2 (**C**; green) also express VSX2/CHX10 (**C** and inset; gray). There are isolated pockets of presumptive RPE cells that co-express MITF (**D** and inset; green) and PAX6 (**D** and inset; red). (**E–H)** After 6–8 weeks, organoids are multilayered structures (**E** and inset) that continue to express SOX2 (**E**; green). Some organoids begin to develop pockets of photoreceptor precursor cells within neural rosette-like structures that express OTX2 (**F**; gray), independently of PAX6 (**F**; red). Areas of pigmented cells that express MITF (**G** and inset; green) independently of PAX6 (**G**; red) are also observed. There is a subpopulation of cells that express the neuronal factor, HuC/D (**H**; green), an early marker of amacrine and ganglion cells and cell proliferation continues at a high rate (**H**; Ki67; red). (**I–L)** After 9–10 weeks, 3D organoids begin to look morphologically similar to a mature retina (inset of **I**) and in some cases have a laminated structure with an outer layer of MITF-positive, RPE cells (**I**; green) with underlying PAX6-expressing cells (**I**; red) and clusters of OTX2-positive photoreceptor precursor cells (**I**; gray). Within neural rosettes, SOX2-positive cells (**J**; green) now lack VSX2/CHX10 (**J** and inset; gray) and have fewer Ki67-positive cells (**J**; red). OTX2 (**K**; gray) is robustly expressed independently of PAX6 (**K** and inset; red) throughout neural rosettes that are now more widespread and larger in size (**K**). Many cells throughout the organoids are now positive for the pan neuronal marker, TUJ1 (**L**; red), but do not yet express the rod photoreceptor-specific factor, NRL (**L** and inset; gray). Small panels to the right of **B** show individual fluorophores. Scale bars = 50 μm, except for **D** = 100 μm.

To demonstrate the utility of this protocol for the cGMP production of photoreceptor precursor cells, we performed immunocytochemical analysis targeted against markers of retinal and photoreceptor precursor cell development. After 3–5 weeks of differentiation (i.e. before retinal cup excision), 3D spheres with evaginated loops of polarized neural epithelia that resemble early eyecup-like structures were readily identified (Fig. 2A and inset). These early F-actin-positive structures were highly organized and comprised of actively proliferating, Ki67-positive cells (Fig. 2A). At this time point, these structures also expressed the early retinal-specific progenitor cell marker SOX2 (Fig. 2B,C), the master eye transcription factor PAX6 (Fig. 2B,D) and the photoreceptor precursor cell-specific transcription factor OTX2 (Fig. 2B). Likewise, the early retinal development marker VSX2/CHX10 was also expressed (Fig. 2C)^25^. By this time point, pigmented presumptive RPE cells began to appear. These cells expressed the RPE-specific transcription factor MITF along with PAX6 (Fig. 2D and inset)^48^.

After 6–8 weeks of differentiation, (i.e. after retinal cup excision) the organoids had thickened and continued to express SOX2 (Fig. 2E and inset). At this stage, clusters of photoreceptor precursor cells that express OTX2 independent of PAX6 began to develop (Fig. 2F). At the site of excision, many organoids maintained pigmented areas of differentiating RPE cells that continued to express MITF (Fig. 2G and inset). Likewise, segregated pockets of HuC/D-positive inner retinal amacrine- and ganglion cell-like neurons were also seen (Fig. 2H). Importantly, after 7 weeks of differentiation, immunocytochemical assessment of 3D organoids from 4 independent patient-specific lines demonstrated complete loss of the pluripotency markers TRA-1-81, TRA-1-60, SSEA-3 and SSEA-4, in contrast to undifferentiated iPSCs (Supplemental Fig. 5).

After 9–10 weeks of differentiation, the organoids begin to resemble mature laminated retina (Fig. 2I, inset) with an outer layer of MITF-positive RPE cells and inner layers of PAX6-positive retinal progenitor and OTX2-positive photoreceptor precursor cells (Fig. 2I). When compared to earlier time points, there was a marked decrease in the number of cells expressing the early retinal progenitor cell marker SOX2 (Fig. 2J) and the cellular proliferation marker Ki67 (Fig. 2J). Likewise, the cells no longer expressed the early retinal transcription factor VSX2/CHX10 (Fig. 2J and inset). As time progressed OTX2-positive photoreceptor precursor cells became more abundant and began to assemble into neural rosette-like structures, clearly segregated from remaining PAX6-positive retinal progenitors (Fig. 2K and inset). Cells throughout the retinal cup expressed the pan-neuronal marker, TUJ1 (Fig. 2L), but did not yet express the rod photoreceptor precursor cell-specific transcription factor NRL (Fig. 2L and inset).

**Figure 3:**
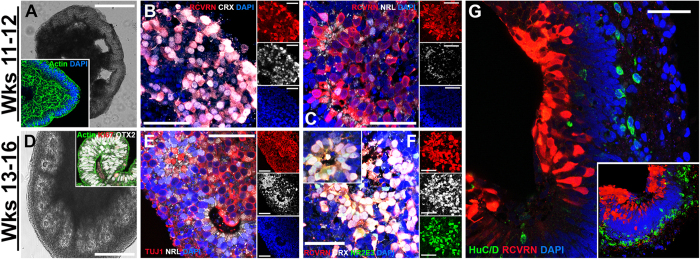
Development of clinical-grade photoreceptor precursor cells. Morphological and immunocytochemical analysis of 3D organoids after 11–12 and 13–16 weeks of differentiation. (**A–C)** After 11–12 weeks of differentiation, organoids have many areas of retinal-like folds (**A**; light micrograph) and retain highly organized F-actin-positive structures (inset in **A**; green). Organoids express the phototransduction molecule, recoverin (**B**; red) and the photoreceptor precursor-specific transcription factor, CRX (**B**; gray), a marker of committed photoreceptor precursor cells that is functionally downstream of OTX2 (Fig. 2). Some recoverin-positive cells within organoids at this stage also begin to express NRL, a rod photoreceptor-specific transcription factor (**C**; gray). Recoverin labeling can also be observed along neuronal-like processes of photoreceptor precursor cells. (**D–G)** Organoids after 13–16 weeks of differentiation are typically full of neural rosettes (**D**; light micrograph) and the majority of OTX2-positive cells (inset of **D**; gray) are now post-mitotic, Ki67-negative cells (inset of **D**; red). By this stage of development, many neuronal cells that are TUJ1-positive (**E**; red) are now positive for NRL (**E**; gray). Many recoverin-positive cells (**F** and inset; red) within neural rosettes co-express NRL (**F** and inset; gray) and NR2E3 (**F** and inset; green), which is functionally downstream of NRL in rod photoreceptor development and is transcriptionally regulated by NRL. Some organoids retain their lamination and resemble mature retina with independent layers of recoverin-positive photoreceptors (**G**; red) and HuC/D-positive inner retinal amacrine- and ganglion-like cells (**G**; green). 3D reconstrutction of a z-stack through a laminated eyecup further demonstrates the separate layers of photoreceptor-like cells and inner retinal neuron-like cells (**G**, inset). Small panels to the right of (**B**,**C**,**E**,**F**) show individual fluorophores. Scale bars **A**,**D** = 400 μm; **B**,**C**,**E**–**G** = 50 μm.

After 11–12 weeks of differentiation, the majority of the retinal cups contained multiple polarized, F-actin-positive neural rosettes (Fig. 3A and inset). It is at this stage that photoreceptor precursor cell-specific markers begin to be expressed and cells commit to becoming mature photoreceptor precursors. This is evident by the initial expression of the photoreceptor-specific cone-rod homeobox protein, CRX and the phototransduction protein, recoverin (RCVRN) (Fig. 3B). CRX is expressed early in post-mitotic photoreceptor precursor cells and is functionally downstream of OTX2^49^,^50^. At the 11–12 week stage, the rod photoreceptor precursor cell-specific marker NRL was also detected (Fig. 3C). Unlike RCVRN, which is detected throughout the entire photoreceptor cell, NRL was restricted to photoreceptor precursor cell nuclei (Fig. 3C).

After 13–16 weeks of differentiation, retinal cups were predominantly comprised of mature OTX2-expressing photoreceptor precursor cells (Fig. 3D), which had exited the cell cycle (i.e. were Ki67-negative) (Fig. 3D, inset). At this time point, TUJ1-positive cells within neural rosettes expressed NRL, which acts in concert with CRX to induce the transcriptional activation of mature rod-specific genes (Fig. 3E)^51^,^52^,^53^. In particular, the rod photoreceptor-specific factor NR2E3 (nuclear receptor subfamily 2, group E, member 3), which is functionally downstream of and transcriptionally controlled by NRL, was also detected at this time (Fig. 3F and inset)^54^. NR2E3 acts to suppress the expression of cone-specific genes thereby irreversibly committing NRL-expressing cells to a rod photoreceptor cell fate^55^. Late in development, some organoids could be identified that retained their lamination and were strikingly similar to mature neural retinas (Fig. 3G and inset). As shown in Fig. 3G, these laminated retinal cups exhibited a RCVRN-positive layer of photoreceptor cells that extended processes towards inner retinal layers comprised of HuC/D-postive presumptive amacrine and retinal ganglion cells (Fig. 3G and inset). Collectively, these data demonstrate that under cGMP conditions we are able to generate clinical-grade, patient-specific, iPSC-derived, eyecup-like structures that contain post-mitotic photoreceptor precursor cells. Cells at this stage have been previously shown to be optimal for transplant-based photoreceptor cell replacement^4^,^7^,^8^,^9^,^10^,^11^.

To determine the efficiency of retinal organoid formation, the number of spheres that developed retinal epithelial protrusions, which were subsequently suitable for manual dissection and expansion at 30-days post-differentiation, was calculated. For this experiment 6 independent patient-specific cell lines were analyzed and the differentiation experiment was repeated 2 independent times. On average 50.17% (SEM 11.53%) of the spheres generated contained retinal epithelium suitable for isolation and expansion. That said, as shown in Supplemental Fig. 4 (each bar represents a single iPSC line and each error bars represents results from repeat differentiations) significant inter-line and inter-experiment variability was detected. With the removal of line 3, which repeatedly produces an atypically high number of retinal organoids, the average was reduced to 29.63%.

### Enhanced S-cone syndrome patient-specific iPSCs demonstrate prototypic lineage specification

**Figure 4:**
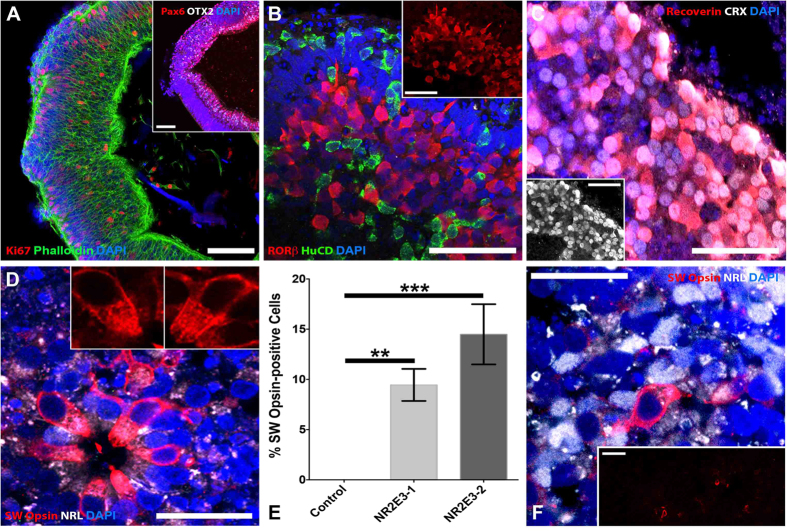
cGMP patient-specific organoids recapitulate photoreceptor cell lineage. Immunocytochemical analysis of 3D organoids from a patient with mutations in *NR2E3* and a patient with normal *NR2E3* alleles. (**A**) *NR2E3* organoids develop normally and are initially indistinguishable from control organoids (Fig. 2). At 5–7 weeks post-differentiation *NR2E3* organoids contain polarized (Phalloidin; green) neural epithelial cells that express PAX6 (inset; red) and OTX2 (inset; gray) and are actively proliferating (Ki67; red). (**B)** After 13 weeks of differentiation, *NR2E3* organoids have cells that express the inner retinal-specific marker, HuC/D (green) and the early photoreceptor-specific lineage transcription factor, RORβ (red and inset). (**C**) After 15–16 weeks of differentiation, presumptive photoreceptors within *NR2E3* mutant organoids are CRX- (gray and inset) and recoverin-positive (red). (**D**) Also around 15–16 weeks, *NR2E3* organoids show robust labeling for SW Opsin, which is specific for blue (S) cones (**D** and insets; red). These SW Opsin-positive cells are NRL-negative (**D** and insets; gray) and have strands of SW Opsin emanating from the cell nucei towards outersegment-like cone-shaped membranes (**D**; insets). (**E)** Quantification of the percentage of SW Opsin-positive cells in two patients with *NR2E3*-associated enhanced S-cone syndrome (NR2E3-1 vs Control; p < 0.01 and NR2E3-2 vs Control; p < 0.001) compared to an unaffected control eyecup at 15–16 weeks post-differentiation. (**F**) Age-matched organoids from a patient with normal *NR2E3* alleles are not positive for SW Opsin (**E**). Further, an eyecup from an individual with normal *NR2E3* alleles after 21 weeks of differentiation has only a few SW Opsin-positive cells (**E** and inset; red) amongst many NRL-positive presumptive rod photoreceptor cells (**E**; gray). Scale bars: **A** and inset of **E**,**F** = 100 μm; all others = 50 μm.

As indicated above, NR2E3 is a photoreceptor precursor cell transcription factor involved in the regulation of photoreceptor cell fate. In particular, during photoreceptor cell development, NR2E3 acts to suppress expression of cone-specific genes, thereby tipping the developmental scale in favor of rod photoreceptors^55^. Mutations in *NR2E3* give rise to the autosomal recessive disease enhanced S-cone syndrome. Individuals with this disease typically have an increased sensitivity to blue light (caused by an increased number of the short wavelength primordial blue (S)-cones), night blindness (due to a lack of or decreased number of rod photoreceptor cells), and varying degrees of green and red sensitivity (due to variations in the number of medium wavelength (M)- and long wavelength (L)-cones developed). To further demonstrate that our cGMP 3D differentiation protocol drives cells down the prototypical photoreceptor cell development pathway, iPSCs derived from an individual with molecularly confirmed enhanced S-cone syndrome (homozygous for *NR2E3* IVS2-2 A > C), were differentiated (Fig. 4)^55^,^56^. Since *NR2E3* is expressed later in development, after *OTX2*, *CRX* and *NRL*, we first wanted to determine whether *NR2E3* mutant organoids display normal early retinal development. Similar to the results shown in Fig. 2, after 5–7 weeks of differentiation, organoids from the *NR2E3* patient contained actively proliferating polarized neural epithelial cells that expressed the retinal progenitor cell-specific factors, PAX6 and OTX2 (Fig. 4A). After 13 weeks of differentiation, *NR2E3* organoids contained cells that expressed the inner retinal neuron-specific marker, HuC/D and the photoreceptor-specific lineage transcription factor, RORβ (Fig. 4B). Similar to organoids shown in Fig. 3 above, after 15–16 weeks of differentiation presumptive photoreceptors within *NR2E3* mutant organoids expressed the photoreceptor-specific transcription factor CRX, which is functionally downstream of RORβ^57^, as well as recoverin (Fig. 4C and inset). Interestingly, at this time point, *NR2E3* organoids also showed robust labeling for SW Opsin (blue cone opsin) (Fig. 4D and inset**s**). These SW Opsin-positive cells were negative for the rod photoreceptor-specific transcription factor, NRL and had visible strands of SW Opsin within outer-segment-like, cone-shaped membranes. To demonstrate that this is indeed an *NR2E3*-associated developmental phenotype, we assessed SW Opsin labelling in organoids differentiated from a similarly aged individual with a normal *NR2E3* gene. After 13–16 weeks of differentiation normal *NR2E3* organoids completely lacked SW Opsin-positive staining compared to organoids derived from two independent patients with *NR2E3*-associated enhanced S-cone syndrome (Fig. 4E (NR2E3-1 vs Control; p < 0.01 and NR2E3-2 vs Control; p < 0.001). A non-*NR2E3* disease control eyecup did not display SW Opsin-positive cells until 21 weeks of differentiation (Fig. 4F and inset). Even at this later stage, there were far fewer SW Opsin-positive cells in the normal *NR2E3* organoids (Fig. 4F and inset). Taken together, these data demonstrate that our cGMP 3D differentiation protocol induces prototypic photoreceptor cell lineage progression and that mutations in key transcriptional regulators, such as *NR2E3*, alter photoreceptor cell fate decisions just as they do *in vivo*.

### Clinical-grade iPSC-derived organoids are non-tumorigenic

One of the major concerns associated with transplantation of pluripotent stem cell-derived products is the risk of teratoma formation after injection. To mitigate this risk, several groups, including our own, have used FACS or bead-based immunopanning to separate the pluripotent tumor-forming cell population from the desired cell type^7^,^11^,^13^,^30^,^37^. Unfortunately, as indicated in the introduction, these approaches often induce extensive cell death and require multiple rounds of selection to obtain acceptable purity. For example, when using a heterogeneous 2D differentiation system, we found that to completely prevent tumor formation after transplantation, multiple rounds of immunopanning with antibodies directed against cell surface pluripotency antigens were required^7^. Unfortunately, with each round of selection there was a significant drop in cell number and viability^7^. As noted above, one of the greatest benefits of the 3D differentiation system is the ability to positively identify, isolate and culture differentiating neural retina. Unlike the 2D system, in which adherent undifferentiated cells often persist, after just 7 weeks of 3D differentiation, the pluripotency markers TRA-1-81, TRA-1-60, SSEA-3 and SSEA-4 were no longer detectable (Supplemental Fig. 5). To demonstrate that retinal cells generated under cGMP conditions using the above described cGMP-compliant protocols were not pluripotent and as such unable to form tumors after transplantation, iPSC-derived organoids were dissociated and injected into immune compromised SCID mice (N = 8 mice, 4 male and 4 female per patient specific cell line; 1–5 × 10^6^ cells/animal). For this study iPSCs from 6 separate patients ranging from 10 to 72 years of age were selected (Supplemental Table 1). Twelve weeks after injection, none of these 48 mice showed any signs of tumor formation. Likewise, there was no evidence of infection or systemic immune reaction, as determined by lack of injection site reaction, maintenance of normal body weight, normal appetite and lack of vocalization upon handling. To demonstrate that this result was not time dependent, an additional cohort of animals was injected for long-term survival studies (N = 8, 4 male, 4 female) (Supplemental Table 1). At 12 months after injection, none of the 8 mice have developed tumors or other systemic issues related to the injection. Moreover, 10 SCID mice that received subretinal injections of dissociated and suspended iSPC-derived eyecup cells displayed a complete lack of intraocular tumors when examined at 3 months post-injection. Collectively, these data demonstrate that by using the cGMP protocols, reagents, and resources described above, we can efficiently generate patient-derived photoreceptor precursor cells.

## Discussion

The neurosensory retina, like the cerebral cortex, is a highly organized laminated structure. The innermost layers of the retina contain, among other cell types, retinal ganglion cells and bipolar interneurons, which are responsible for first order visual processing and relaying of visual information from the light sensing photoreceptor cells of the outer retina, to the brain. Dysfunction and eventual death of photoreceptor cells is the primary cause of vision loss associated with retinal degenerative disorders such as retinitis pigmentosa. Extensive clinical and histopathologic data reveal that the inner retina is relatively spared in many retinal degenerative diseases, even at advanced stages^1^,^2^,^58^,^59^,^60^,^61^, which raises the exciting possibility that one might be able to restore vision to patients with retinal degenerative blindness by transplanting new outer retinal cells into the subretinal space. Using existing surgical strategies for subretinal surgery, stem cell-derived photoreceptor cells can be placed immediately adjacent to their synaptic targets, the remaining host bipolar neurons. This physical proximity coupled with the lack of inhibitory myelin-associated proteins in the retina creates a situation in which newly transplanted photoreceptor cells need only extend their axons relatively short distances through an environment that, compared to other CNS compartments, is highly conducive to growth and synaptic integration.

In this study, we derived clinical-grade patient-specific iPSC-derived photoreceptor precursor cells in a non-profit, academic cGMP facility. Specifically, under ISO class 5 conditions, we developed and tested methods for the isolation and expansion of patient dermal fibroblasts, generation of patient-specific iPSCs and 3D differentiation of photoreceptor precursor cells. Unexpectedly, the ability to isolate and expand dermal fibroblasts from 3 mm punch biopsies, a seemingly trivial aspect of this study, was significantly influenced by patient age. In our hands, all of the commercially available cGMP-compatible cell culture media evaluated were inadequate for isolation and expansion of cells from all but our youngest donors. As many of the patients with advanced retinal degeneration are over 55 years of age, new cGMP-compliant reagents needed to be developed. Using these new media, dermal fibroblasts suitable for iPSC generation were successfully isolated and expanded from 35 separate patients with inherited retinal degenerative disease with an average age of 57. The ability to generate photoreceptor precursor cells from any individual regardless of age is essential if one aspires to use the iPSC technology to treat all forms of retinal degeneration.

At least two fairly significant and interrelated obstacles remain to be overcome before stem cell-based photoreceptor transplantation can be widely used to treat human retinal disease: the cost of producing transplantable cells and the potential for post-transplantation immunologic injury to the cells and surrounding host tissues. The most financially feasible way to manufacture transplantable cells would be to employ a single cell source that could be extensively validated, grown in large quantities, and then used to treat a very large number of patients regardless of genotype. Unfortunately, such single source cells would be a poor immunologic match for most patients and a large body of clinical evidence suggests that the subretinal space of patients with advanced retinal degeneration is freely accessible to the immune system^62^,^63^,^64^. As a result, a single cell source strategy would likely require long-term immunosuppression to allow the transplanted cells to survive and avoid immune-mediated injury to the adjacent host tissues.

At the other extreme, one could use induced pluripotent stem cell (iPSC) technology to create transplantable cells from the skin of the patient for whom the transplant is intended, and by so doing create a perfect immunologic match. In this way, one could avoid or at least greatly minimize the need for significant long-term immunosuppression. However, with this iPSC strategy, one would need to create and characterize a new cell line for each patient, and in many cases, also correct the patient’s disease-causing genetic defect using some type of genome editing approach such as the CRISPR/Cas9 system^65^,^66^,^67^,^68^.

At the present time, the degree to which an average patient can tolerate some immunological mismatching between themselves and their transplant is unknown. The concern is that an immunologic reaction to an unmatched transplant might not only injure the transplant itself, but could also injure the patient’s remaining bipolar cells and ganglion cells thereby diminishing the likelihood that a future transplant attempt would succeed. One could reduce the risk that a transplant-induced immune response would completely blind a sighted patient by restricting the treatment to the poorer of a patient’s two eyes. Although safer, this strategy would not completely eliminate the risk. Sympathetic ophthalmia is a rare disease in which surgery or injury to one eye incites an immune reaction that causes an inflammatory injury to the patient’s contralateral or sympathetic eye^69^,^70^. Despite aggressive immune suppression, some cases of sympathetic ophthalmia can result in irreversible blindness^71^.

The iPSC advantage of a perfect immunologic match would be difficult to deliver on a population-wide scale. For example, using existing technology, a single individual working in a single cGMP suite could produce, characterize and differentiate no more than a handful of patient-specific cell lines per year. Genome editing of the disease-causing mutations would reduce this number further, even if the mutations were common and the reagents necessary for the editing had already been designed and optimized. The cells would need to be grown in a completely xeno-free environment because the way in which the cells are treated *in vitro* can have a clinically meaningful effect on immunogenicity. For instance, exposing autologous cells to animal-derived products such as fetal bovine serum has been shown to induce antigenicity and result in severe immune reaction following transplantation^72^,^73^.

There are a number of ways that the gulf between a single cell source strategy and a patient-derived iPSC strategy could be narrowed. To increase the number of patients that could be treated using patient-specific iPSCs, one could train a large number of highly skilled personnel and develop a network of regional cGMP facilities for them to work in. Such an approach would carry the risk of significant inter-technician and inter-site variability. Alternatively, robotic technologies, such as those recently described by Paull *et. al.*^74^, have the potential to significantly reduce hands on time and increase the number of cell lines that can be simultaneously processed by a single individual. This would not only reduce cost, it would also increase technical reproducibility, a critically important aspect of clinical scale up.

Another way to reduce the total number of cell lines needed to treat all retinal degeneration patients while still attempting to achieve some degree of immunologic matching is to create a series of cell banks that contain a limited number of fully validated lines with common HLA haplotypes, designed to match a significant portion of the patient population. Such “super donor” cell banks can be produced by generating iPSCs from patients with selected haplotypes^75^,^76^ or by genetic manipulation of HLA genotypes^76^. However, to cover all possible HLA combinations, an extraordinarily large cell bank would be required^76^ which would offer little financial advantage over an autologous iPSC approach. Any financially feasible deployment of this strategy would likely fail to cover a sizable portion of the general population. Another idea that is being aggressively pursued by a number of groups is the production of a “universal donor”, a cell type that would be immunologically silent regardless of the patient’s genotype. For instance, Riolobos and colleagues recently demonstrated that the targeted deletion of *Beta-2 Microglobulin*, a known transporter of HLA antigens, resulted in an ES cell line that does not express any class I HLA surface antigens^76^. When tested under conditions of limited HLA class II expression, these cells were shown to be immunologically silent^76^. However, to date, no one has yet produced ESCs or iPSCs that are completely devoid of immunologic identifiers and thus it remains to be determined whether the universal donor approach will be clinically useful.

Regardless of the origin of the cells, the clinical-grade cGMP-compliant reagents and procedures described here have the potential to be widely applicable. The 3D differentiation protocol in particular can be used to generate photoreceptor precursor cells regardless of whether unmatched, super donor, universal or patient-specific pluripotent stem cells are utilized. A remarkable aspect of this, and of other published 3D retinal differentiation protocols^13^,^17^,^21^,^25^, is the degree to which they accurately recapitulate human retinal development. Although the majority of organoids generated using this protocol develop outer nuclear layers that consist of photoreceptor cells tightly packed within neural rosettes, a subset of the organoids maintain a polarized laminated architecture reminiscent of the mature human retina. It is fortuitous that following excision and manual isolation, organoids continue to develop such that photoreceptor cells lie in the outermost layers of the spheres where they receive a constant supply of nutrients and oxygen that are required for normal health and development. As demonstrated by Yoshiki Sasai and colleagues^17^,^28^, as organoids grow, their centers become hypoxic and some inner retinal neurons are lost. This would be problematic if the goal were to generate and maintain mature inner retinal neurons but is actually beneficial when attempting to generate cells for photoreceptor cell replacement. That is, the majority of cells that remain after 90–120 days of differentiation are post-mitotic photoreceptor precursor cells, which are at the optimal stage of retinal development for retinal transplantation. This method of self-selection negates the need for FACS or bead-based cell enrichment methods. That is, after the organoids are generated, they can be readily dissociated and delivered to the clinic for subretinal injection or loaded onto cell support scaffolds for further maturation and subsequent subretinal transplantation.

### Summary

In summary, in this study we have used open source components to develop cGMP-compliant protocols, reagents and infrastructure that can be used by any group to produce clinical-grade patient-specific iPSCs and in turn, photoreceptor precursor cells suitable for the treatment of retinal degenerative blindness. Although a patient-specific iPSC strategy is significantly costlier than a single cell source approach, the reduction of immunologic stimulus it affords may be warranted until the immunology of subretinal transplantation is more completely understood.

## Additional Information

**How to cite this article**: Wiley, L. A. *et al*. cGMP production of patient-specific iPSCs and photoreceptor precursor cells to treat retinal degenerative blindness. *Sci. Rep.* **6**, 30742; doi: 10.1038/srep30742 (2016).

## Supplementary Material

Supplementary Information

## Figures and Tables

**Table 1:** Acceptance criteria for release and use of a product in the Dezii Translational Vision Research Facility.

cGMP Release criteria		
Parameter	Method	Specification
CMV, EBV, HAV, HBV, HCV, HHV-6, 7&8, HIV 1&2, HPV, HTLV 1&2)	PCR/qPCR	Negative
Retroviruses	Q-PERT	<5.0 × 10^−7^ U ml^−1^
SFV, SRV, SLTV, SV40	PCR/qPCR	Negative
BVDV, MRV1, 2&3, Rabies, BTV, BAd, BPV, BRSV	PCR/qPCR	Negative
PAd, PPV, TGEV, PHE-CoV	PCR/qPCR	Negative
Sterility testing		
Mycoplasma	qPCR	Negative
Fungus/Bacteria and Bacteriostasis/Fungistasis	Outgrowth assay (USP<71>)	No growth

**Table 2:** Acceptance criteria for cGMP release.

cGMP Release criteria		
Parameter (iPSCs)	Method	Specification
Karyotyping	Giemsa Stain	Normal
Score Card	qPCR based assay	positive self renewal, negative endodermal, mesodermal, and ectodermal lineage
Genetic Markers	PCR based assay	
Transcript	rt-PCR	Positive self renewal markers
Mutational burden	Whole Genome Sequencing	No variations in retinal, cell cycle or cancer genes
Mycoplasma	Quantitative PCR	< assay detection limit of 10 copies/PCR reaction
**Parameter (Retinal Organoids)**	**Method**	**Specification**
Identity	ICC	Positive photoreceptor precursor, negative self renewal
Genetic Marker QC	PCR based assay	Matches known marker genotypes of patient
Mycoplasma	qPCR	Negative
Sterility: Fungus/Bacteria and Bacteriostasis/Fungistasis	Outgrowth assay (USP <71>)	No Growth

**Table 3:** Patient cohort used to test commercially available FibroGRO fibroblast cell growth media.

Patient	Age	Sex	FibroGRO™
T3	29	Male	++
T2	36	Male	+
T1	47	Male	+
T7	56	Male	−
T4	61	Male	−
T6	79	Female	−
T5	81	Male	−

Age = age at biopsy; ++ = by 14-days post-platting a sufficient number of dermal fibroblasts for iPSC generation were present; + = by 14 days post-plating dermal fibroblasts were present however, they were sparse and not sufficient for iPSC generation; −at 14-days post-platting few if any dermal fibroblasts were present.

**Table 4:** Patient-specific skin biopsies obtained and cultured under cGMP conditions using in house developed xeno-free fibroblast culture media (IxMedia).

Biopsy #	Age	OD VA	OS VA	Clinical Diagnosis	Gender	Molecular Diagnosis
DB-001	72	20/100	20/100	Retinitis Pigmentosa	Male	MAK
DB-002	86	20/40	20/100	Age Related Macular Degeneration	Male	CFH, ARMS2
DB-003	28	HM	LP	Leber Congenital Amaurosis	Male	None
DB-004	79	20/100	20/80	Retinitis Pigmentosa	Male	USH2A
DB-005	47	NLP	NLP	Retinitis Pigmentosa	Female	BBS1
DB-006	32	NLP	NLP	Retinitis Pigmentosa	Male	PCDH21
DB-007	81	NLP	LP	Retinitis Pigmentosa	Male	MAK
DB-008	54	NLP	LP	Leber Congenital Amaurosis	Male	RDH12
DB-009	57	20/1000	LP	Retinitis Pigmentosa	Female	RHO
DB-010	69	LP	LP	Retinitis Pigmentosa	Male	BBS1
DB-011	67	NLP	LP	Retinitis Pigmentosa	Female	PCDH21
DB-012	54	NLP	NLP	Retinitis Pigmentosa	Female	None
DB-013	58	HM	NLP	Leber Congenital Amaurosis	Male	RPGRIP1
DB-014	41	LP	5/350	Retinitis Pigmentosa	Female	CERKL
Db-015	61	20/150	NLP	Usher Syndrome	Male	USH2A
DB-016	65	1/350	LP	Retinitis Pigmentosa	Female	None
DB-017	17	LP	LP	Leber Congenital Amaurosis	Female	None
DB-018	67	LP	LP	X-linked Retinitis Pigmentosa	Male	RPGR
DB-019	41	LP	LP	Usher Syndrome	Male	SLC26A4
DB-020	69	20/60	LP	Choroideremia	Male	CHM
DB-021	67	20/100	LP	Retinitis Pigmentosa	Male	PRPF8
DB-022	57	LP	CF	Leber Congenital Amaurosis	Male	NMNAT1
DB-023	58	LP	LP	Retinitis Pigmentosa	Male	BBS1
DB-024	23	LP	LP	Batten Disease	Female	CLN3
DB-025	37	HM	LP	Leber Congenital Amaurosis	Male	CEP290
DB-026	61	HM	LP	Retinitis Pigmentosa	Male	None
DB-027	58	HM	LP	Retinitis Pigmentosa	Male	USH2A
DB-028	63	LP	LP	Usher Syndrome	Female	USH2A
DB-029	65	HM	20/80	Usher Syndrome	Male	USH1C
DB-030	73	HM	LP	Choroideremia	Male	CHM
DB-031	49	CF	20/50	Usher Syndrome	Male	USH2A
DB-032	45	20/50	20/50	Usher Syndrome	Male	PCDH15
DB-033	47	20/40	20/40	Usher Syndrome	Female	PCDH15
DB-034	71	NLP	NLP	Retinitis Pigmentosa	Male	RPGR
DB-035	61	LP	LP	Retinitis Pigmentosa	Male	RPGR

Age = patient age at biopsy, OD VA = Visual acuity of the right eye, OS VA = Visual acuity of the left eye. HM = hand motion, CF = counts fingers, LP = light perception, NLP = no light perception.
