# Supplementary material for: cGMP production of patient-specific iPSCs and photoreceptor precursor cells to treat retinal degenerative blindness
Source: Sci Rep. 2016 Jul 29;6:30742. doi: 10.1038/srep30742 (PMC4965859; doi:10.1038/srep30742)
Supplement: Supplementary Information [file srep30742-s1.pdf]

Supplementary Information

Title: cGMP production of patient-specific iPSCs and photoreceptor precursor cells to treat retinal degenerative blindness

Running Title: cGMP production of patient-specific photoreceptors

Authors: Luke A. Wiley[1], Erin R. Burnight[1], Adam P. DeLuca[1], Kristin R. Anfinson[1], Cathryn M. Cranston[1], Emily E. Kaalberg[1], Jessica A. Penticoff[1], Louisa M. Affatigato[1], Robert F. Mullins[1], Edwin M. Stone[1] and Budd A. Tucker[1]

Supplemental Table 1. List of clinical-grade patient-specific iPSC-derived photoreceptor precursor cell lines used for *in vivo* tumorigenicity analysis. Age = patient age at biopsy, 12 week endpoint = number of mice analyzed per patient-specific photoreceptor precursor cell line at 12 weeks post-injection, 12 month endpoint = number of mice analyzed per patient-specific photoreceptor precursor cell line at 12 months post-injection. IM = intramuscular, SR = subretinal.

Age	Clinical Diagnosis	Gender	12 week endpoint	12 month endpoint	Injection Site
10	Retinitis Pigmentosa	Male	N=8 (4M, & 4F)	N=8 (4M, & 4F)	IM
38	Retinitis Pigmentosa	Female	N=8 (4M, & 4F)	N=0	IM
47	Best Disease	Female	N=8 (4M, & 4F)	N=0	IM
58	Retinitis Pigmentosa	Male	N=8 (4M, & 4F)	N=0	IM
68	Retinitis Pigmentosa	Female	N=8 (4M, & 4F)	N=0	IM
72	Retinitis Pigmentosa	Male	N=8 (4M, & 4F)	N=0	IM
10	Retinitis Pigmentosa	Male	N=5 (2M, & 3F)	N=0	SR
72	Retinitis Pigmentosa	Male	N=5 (3M, & 2F)	N=0	SR

Supplemental Figure 1

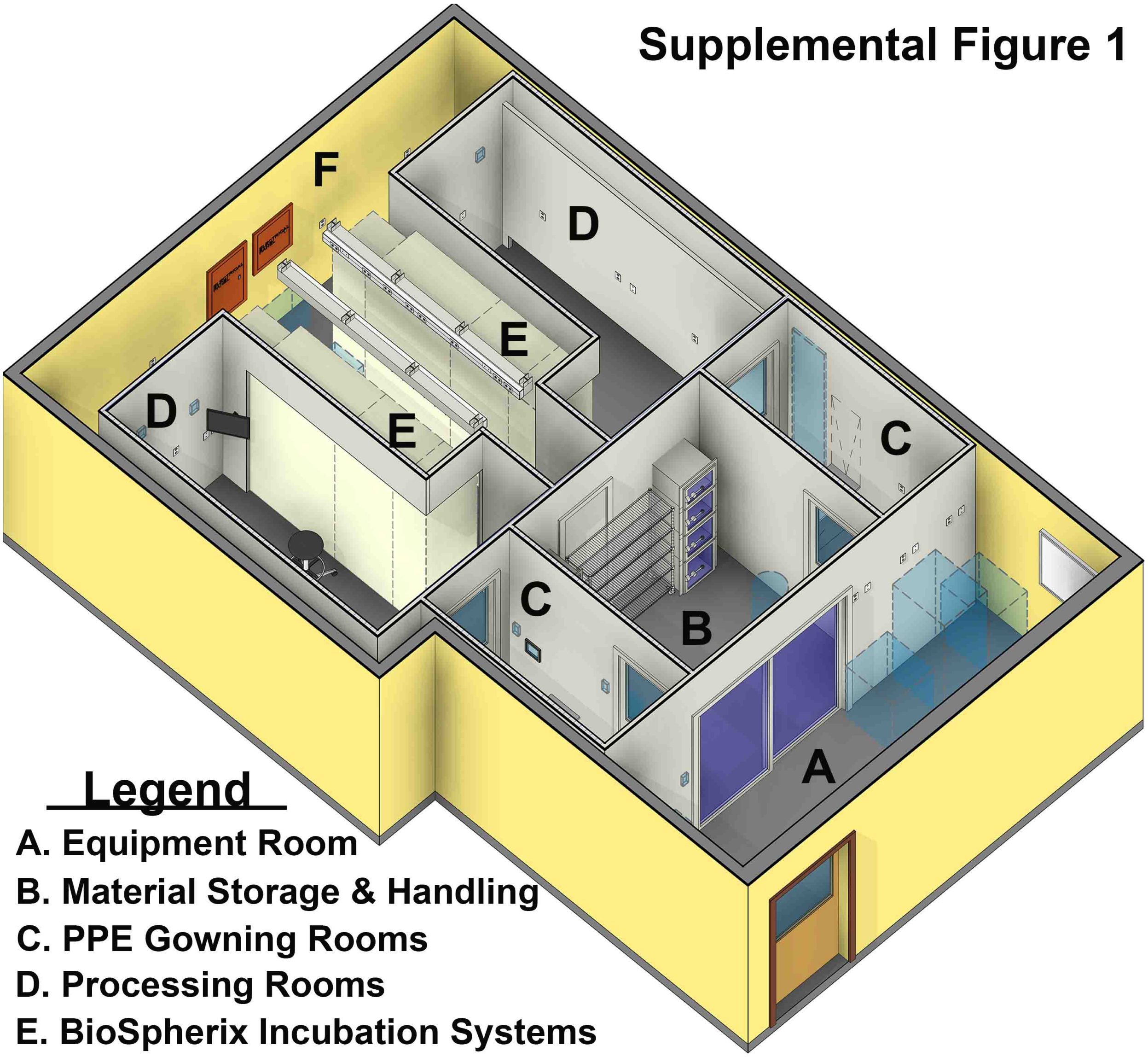

Legend

A. Equipment Room
B. Material Storage & Handling
C. PPE Gowning Rooms
D. Processing Rooms
E. BioSpherix Incubation Systems
F. Service Chase

Supplemental Figure 1: The Steven W. Dezii Translational Vision Research Facility. Architectural rendering of the Steven W. Dezii Translational Vision Research Facility. This state-of-the-art FDA-registered cGMP facility contains a HEPA-filtered equipment room (A), an independent ISO Class 7 material storage and handling room (B), two ISO Class 7 PPE gowning rooms (C), and two ISO Class 6 processing rooms (D) equipped with two ISO Class 5 BioSpherix Xvivo Closed Incubation Systems (E). An independent service chase (F) room serves as access to expunged media and allows for monitoring and maintenance of the BioSpherix Systems.

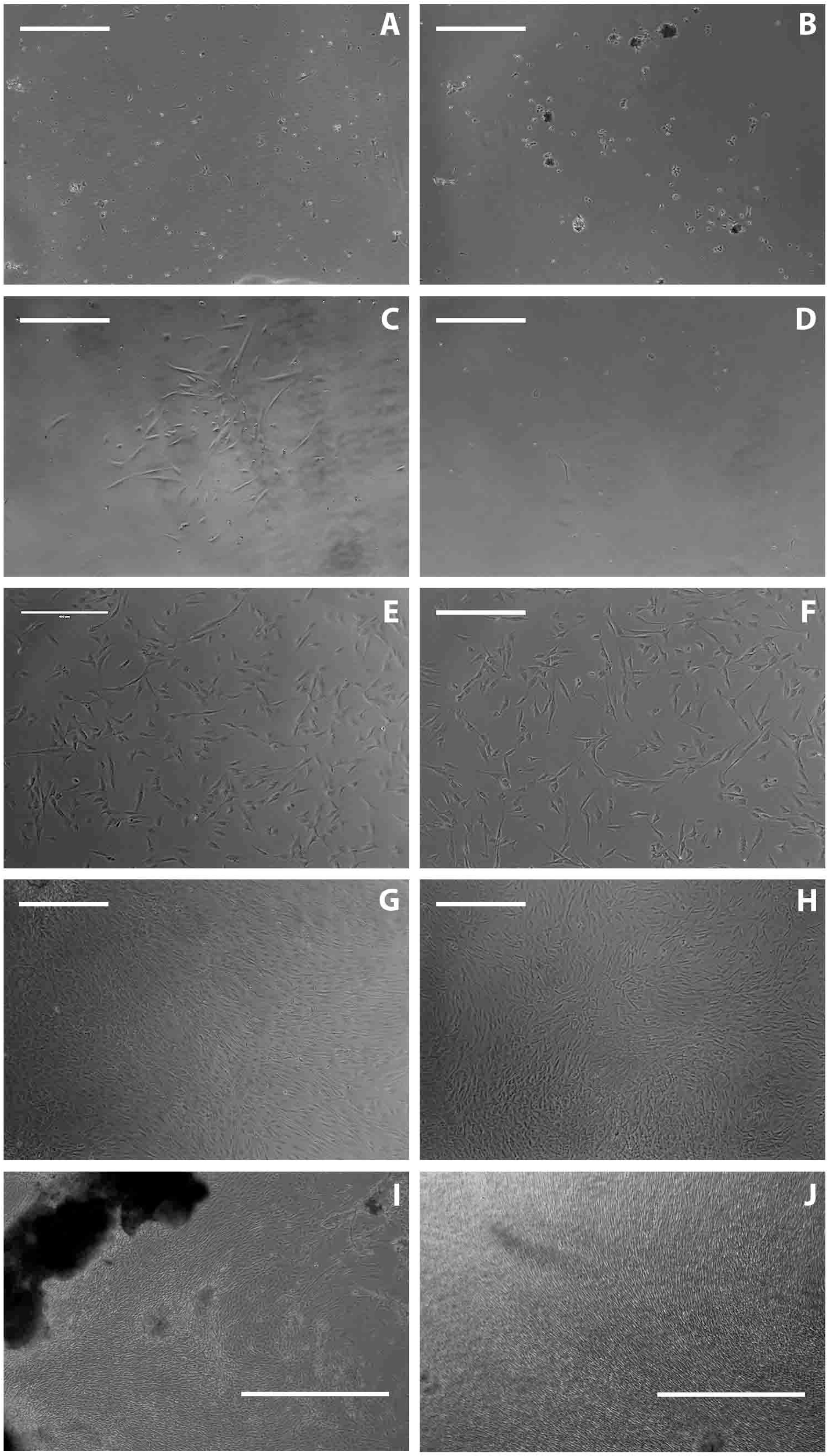

Supplemental Figure 2

Supplemental Figure 2: Testing of commercial xeno-free fibroblast culture medias and in-house-generated cGMP IxMedia. At 10 days post-plating fibroblasts cultured in either FGM-CD™ (A-B) or MesenCult™ (C-D) were sparse, compared to healthy, evenly distributed fibroblasts observed after just a single day of culture in IxMedia (E-F). Ten days after culture in IxMedia, fibroblasts were confluent and ready to be passaged (G-H). Fibroblasts from an 81-year-old patient with no light perception vision that failed to grow when cultured in either FGM-CD™ or MesenCult™ displayed extensive outgrowth after 7 days in IxMedia (I) and were ready for passage by 14 days post-plating (J). Scale bars = 200 µm.

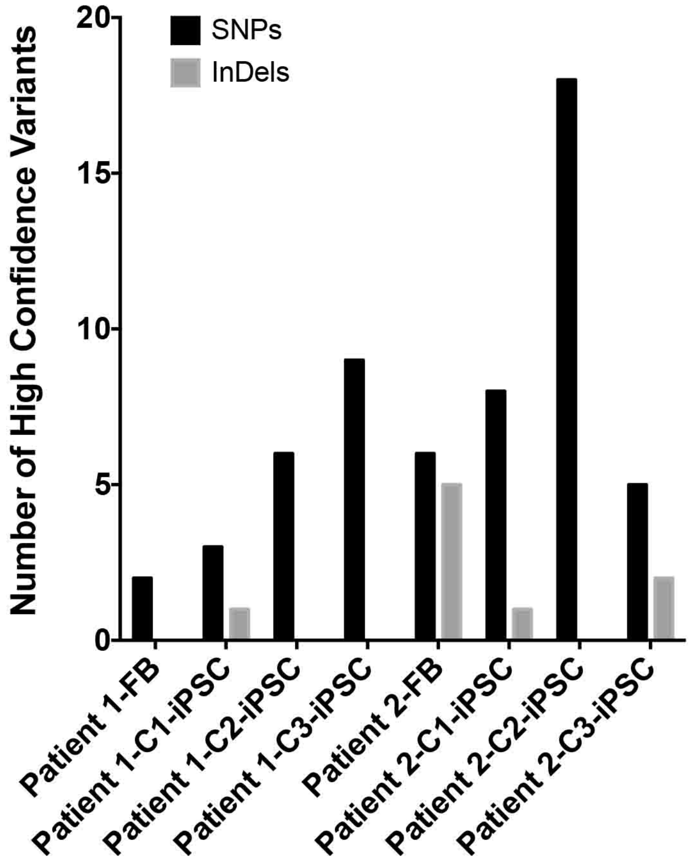

Supplemental Figure 3

Supplemental Figure 3: Whole genome sequencing of patient-derived fibroblasts and iPSCs. Number of high confidence single nucleotide polymorphisms (SNPs) and insertions or deletions (InDels) observed in fibroblasts and three independent iPSC clones derived from Patient 1 and Patient 2 (see Figure 1).

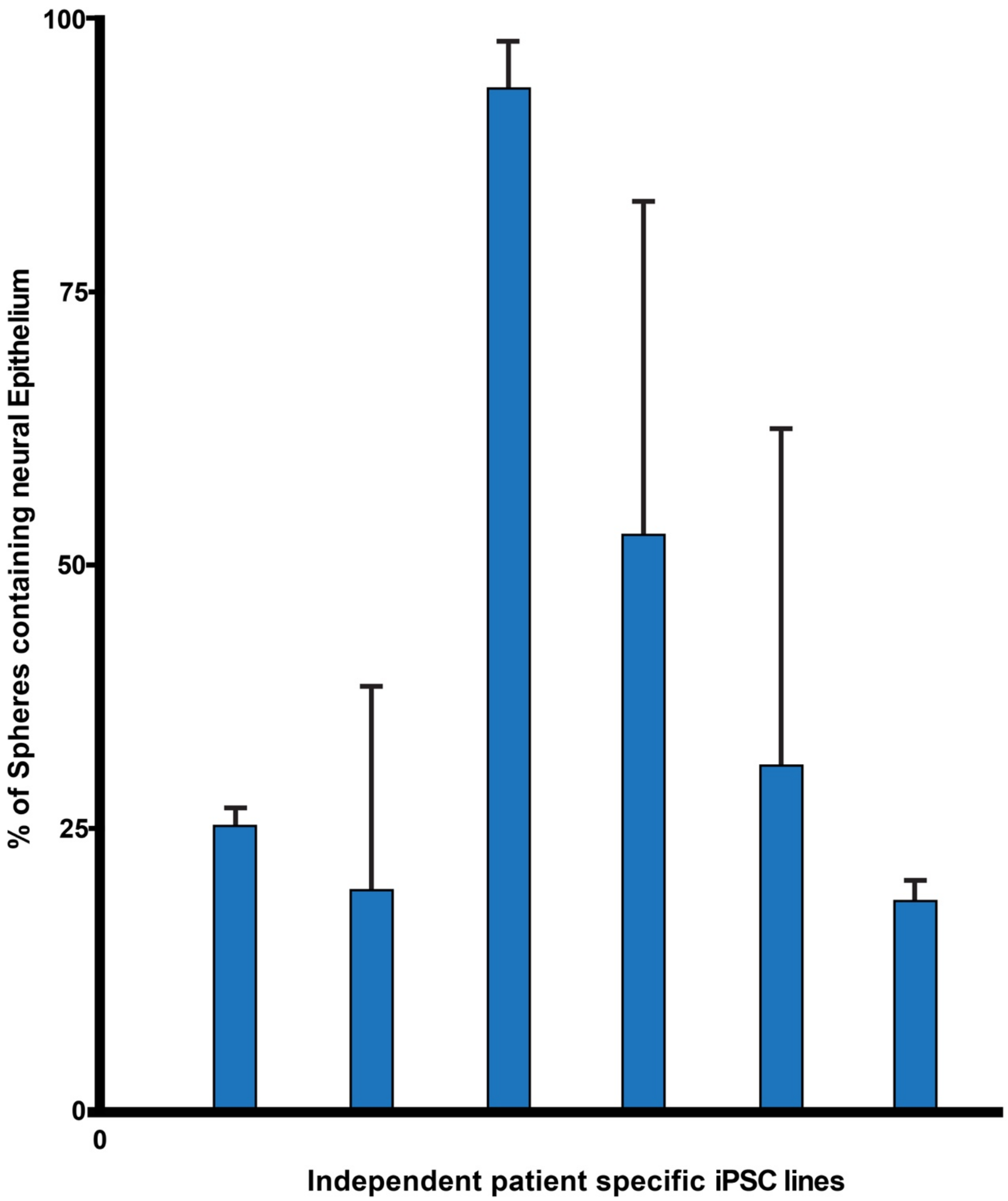

Supplemental Figure 4

Supplemental Figure 4: Efficiency of retinal organoid formation. Each bar represents an independent patient-specific cell line. Error bars – SEM, represent independent experiment repeats.

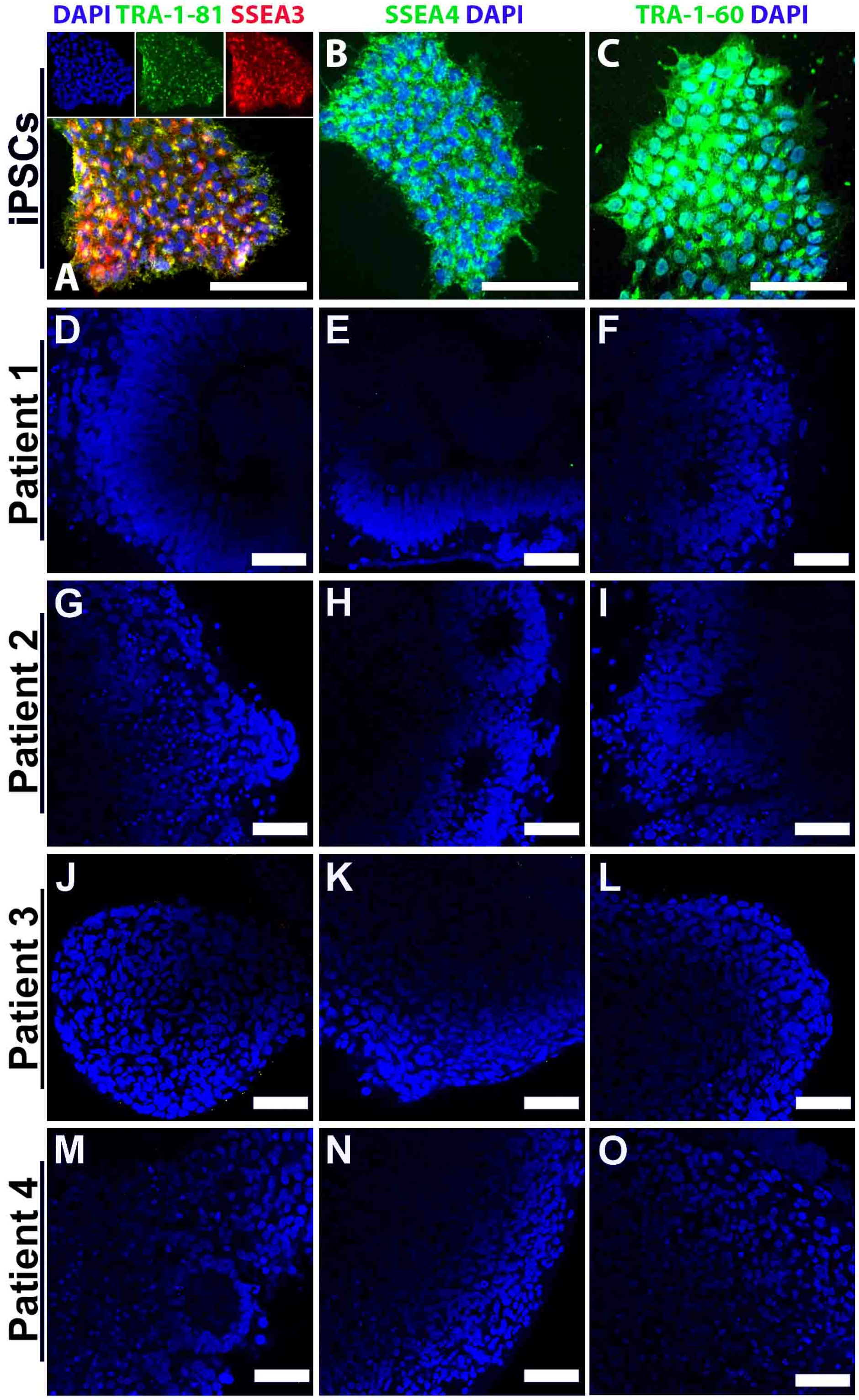

Supplemental Figure 5

Supplemental Figure 5: Differentiating 3D eyecups exhibit complete loss of pluripotency markers. Immunocytochemical assessment of the pluripotency markers, TRA-1-81, TRA-1-60, SSEA-3 and SSEA-4 in undifferentiated iPSCs and 3D eyecups after 7 weeks of differentiation. **A-C:** Undifferentiated iPSCs exhibit positive labeling for the pluripotency markers TRA-1-81 (A; green), SSEA-3 (A; red), SSEA-4 (B; green) and TRA-1-60 (C; green). **D-O:** All four independent lines of patient-specific differentiated 3D eyecups (Patients 1-4) completely lack expression of TRA-1-81, TRA-1-60, SSEA-3 and SSEA-4. Scale bars = 100 μm in A-C and 50 μm in D-O.
